# Tomato Lycopene and Lung Cancer Prevention: From Experimental to Human Studies

**DOI:** 10.3390/cancers3022333

**Published:** 2011-05-11

**Authors:** Paola Palozza, Rossella E. Simone, Assunta Catalano, Maria Cristina Mele

**Affiliations:** 1 Institute of General Pathology, School of Medicine, Catholic University, L. Go F. Vito, Rome 1 00168, Italy; E-Mails: simonerossella@libero.it (R.E.S.); assuntacatalano@libero.it (A.C.); 2 Institute of Biochemistry and Clinical Biochemistry, School of Medicine, Catholic University, L. Go F. Vito, Rome 1 00168, Italy; E-Mail: mcmele@rm.unicatt.it (M.C.M.)

**Keywords:** lycopene, carotenoids, lycopene metabolites, lung cancer prevention, cancer cell proliferation, apoptosis

## Abstract

Increasing evidence suggests that tomato lycopene may be preventive against the formation and the development of lung cancer. Experimental studies demonstrated that lycopene may inhibit the growth of several cultured lung cancer cells and prevent lung tumorigenesis in animal models through various mechanisms, including a modulation of redox status, cell cycle arrest and/or apoptosis induction, a regulation of growth factor signaling, changes in cell growth-related enzymes, an enhancement of gap junction communication and a prevention of smoke-induced inflammation. In addition, lycopene also inhibited cell invasion, angiogenesis, and metastasis. Several lycopene metabolites have been identified, raising the question as to whether the preventive effects of lycopene on cancer risk is, at least in part, due to its metabolites. Despite these promising reports, it is difficult at the moment to directly relate available experimental data to human pathophysiology. More well controlled clinical intervention trials are needed to further clarify the exact role of lycopene in the prevention of lung cancer cell growth. Such studies should take into consideration subject selection, specific markers of analysis, the levels of carotenoids being tested, metabolism and isomerization of lycopene, interaction with other bioactive food components. This article reviews data on the cancer preventive activities of lycopene, possible mechanisms involved, and the relationship between lycopene consumption and human cancer risk.

## 1. Introduction

The human lung, due to the oxidative and ozone stress to which it is exposed, is particularly vulnerable to oxidative damage. The volume of oxygen that passes through the lung is so great that it is likely to be extremely vulnerable unless there are multiple levels of antioxidant protection. The lung operates under a higher oxygen pressure environment than other internal organs. This can result in the conversion of high concentrations of antioxidants into pro-oxidants. In addition, the presence of other oxidants such as ozone can enhance risk of oxidative damage [1]. To protect against these vulnerabilities, a number of antioxidant defences are present in the lung epithelial lining fluids, as well as within the lung cells. Ascorbic acid and tocopherols have been studied in this context [2]. Tocopherols are believed to be, by sheer mass, the first line of defense against membrane oxidation. Ascorbic acid levels are known to be positively related to pulmonary function [3]. Both lycopene and β-carotene have been measured in lung tissue, from autopsy study and from bronchial lavage, lending credence to the possibility that carotenoids are a lung defense mechanism despite the fact that their concentrations are much lower than that of α-tocopherol [3-7]. Evidence from observational epidemiological studies rapidly accumulated and tended to support an inverse association of lung cancer incidence with β-carotene intake and with serum concentrations of β-carotene. This evidence led to the initiation of several large scale randomized chemo-prevention trials to test the hypothesis that β-carotene supplements protected against lung cancer, but those trials had disappointing results. Indeed, β-carotene supplementation actually was found to increase the risk of lung cancer in high-risk populations [8,9]. Whereas high-dose β-carotene supplementation is ineffective in reducing lung cancer risk in randomized trials, many questions remain about the potential benefits of the intake of lower doses of β-carotene over prolonged periods. Furthermore, there is substantial interest in the potential role of other carotenoids in lung cancer prevention.

**Figure 1.**
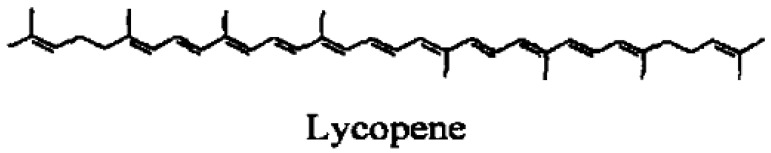
Chemical structure of lycopene.

Among the different carotenoids, one of the ongoing research candidates for the prevention of lung cancer risk is lycopene. Lycopene, one of more than 600 carotenoids synthesized by plants and photosynthetic microorganism [10], is a tetraterpene hydrocarbon containing 40 carbon atoms and 56 hydrogen atoms. Lycopene is the most abundant carotenoid in tomatoes (*Lycopersicon esculentum* L.) with concentrations ranging from 0.9–4.2 mg/100 g depending upon the variety [11]. Tomato sauce and ketchup are concentrated sources of lycopene compared to unprocessed tomatoes [12]. Other edible sources of lycopene include rosehips [13], watermelon, papaya, pink grapefruit, and guava [14]. Belonging to the hydrocarbon carotene class of carotenoids, lycopene is acyclic and contains 11 conjugated and two non-conjugated double bonds (Figure 1). Because it is acyclic and lacks a bionone ring, lycopene has no pro-vitamin A activity. Although dietary lycopene is predominantly all-*trans*, more than 50% of the lycopene present in human serum and tissues is in *cis*-forms [15]. Lycopene absorption from dietary sources is influenced by several factors, such as the break up of the food matrix containing lycopene, cooking temperatures and the presence of lipids and other lipid soluble compounds including other carotenoids [16]. Absorption of lycopene is similar to that of other lipid soluble compounds and is absorbed across the gastrointestinal tract via a chylomicron mediated mechanism. Incorporated into chylomicrons, lycopene and other carotenoids are transported from the intestinal mucosa to the general circulation via the lymphatic system. In the blood, carotenoids are transported by lipoproteins. In particular, LDL is the primary carrier of lycopene. In general, 10–30% of the dietary lycopene is absorbed by humans. It is absorbed equally efficiently from different sources of lycopene including tomato sauce, tomato juice and tomato oleoresin capsules [17]. Liver, seminal vesicles and prostate tissue are the primary sites of lycopene accumulation *in vivo* [18]. Furthermore, Liu *et al.* [19] found that lycopene can be selectively localized to the nuclear membrane and nuclear matrix, suggesting a possible role for a lycopene receptor or transporter. Lycopene in the tissues undergoes oxidation and metabolism. Several oxidized form of lycopene and polar metabolites have been isolated and identified [20]. Apo-6′and apo-8′-lycopenals were reported to be present in raw tomatoes [21]. Some lycopene metabolites, including 5,6-dihydroxy-5,6-dihydro-lycopene [22], and apo-6′-, apo-8′-, apo-10′-, apo-12′- and apo-14′-lycopenals have been also detected in human plasma.

Epidemiological studies show that populations consuming a tomato-rich diet, containing high levels of lycopene, exhibit lower risk of certain types of cancers, including lung cancer [23]. Moreover, increasing experimental studies have proven that lycopene molecule possesses anti-tumoral activity in lung tumorigenesis [24]. The extended conjugated polyene chain of lycopene is an electron-rich system, susceptible to attack by electrophilic reagents. Therefore, carotenoids like lycopene are unstable and highly reactive towards oxygen and free radicals [25]. This reactivity of lycopene is the basis for its anti-oxidant activity in biological systems that might contribute to its efficacy as a chemoprevention agent. Moreover, it has been suggested that lycopene can exert modulatory action on cancer by interacting with a wide spectrum of molecular targets central to the cell signaling machinery [26]. Other mechanisms of chemoprevention by lycopene include the up-regulation of the antioxidant response element leading to the synthesis of cytoprotective enzymes, the enhancement of intercellular gap junction communication [27-29], the modulation of hormonal, inflammatory and immune system [30-33] and metabolic pathways [34-36]. This review summarizes the most current knowledge with respect to lycopene role in the prevention of lung cancer.

## 2. Antitumoral Effects of Lycopene in Lung Cancer Cells

Reactive oxygen species (ROS) and the related oxidative damage have been implicated in the pathogenesis of various human chronic diseases [37-39]. Lycopene is one of the most potent antioxidants [40] and has been suggested to prevent carcinogenesis by protecting critical biomolecules including lipids, proteins and DNA [41,42]. Several studies have indicated that lycopene is an effective antioxidant and free radical scavenger. Lycopene, because of its high number of conjugated double bonds, exhibits higher singlet oxygen quenching ability compared to β-carotene or α-tocopherol [43]. In cell culture, lycopene was shown to inhibit nitration of proteins and DNA strand breakage caused by peroxynitrite treatment of Chinese hamster lung fibroblasts [44]. Oxidative DNA damage caused by the redox-cycling of catechol-estrogens in plasmid DNA and Chinese hamster lung fibroblasts was also reduced by lycopene [45]. In Hep3B cells treated with H_2_O_2_, lycopene was found to reduce DNA damage in a dose-dependent manner as indicated by the comet assay [46].

A number of studies showed that lycopene inhibited the growth of human cancer cells grown in cultures. The growth-inhibitory effects of lycopene were observed not only in lung cancer cells, but also in other cell types, including prostate, breast, hepatoma, stomach, colon and oral cancer cells [30,46-56]. In some studies, lycopene has been reported to be more effective as an anticancer agent than α- or β-carotene [30]. Most studies on cell proliferation with lycopene treatment show that the carotenoid induced a cell cycle arrest at the G1 phase and that such an effect increased with the dose of lycopene [47-56]. Very few have reported no effect on inhibition of cell proliferation, but effect on apoptosis induction from lycopene treatment [55-58]. Moreover, lycopene has been shown to inhibit tumor metastasis *in vitro* [59-61].

A limitation of cell culture studies is the extremely hydrophobicity of lycopene, which is an obstacle for conducting cell culture studies. Since lycopene is insoluble in water, steps must be taken to enhance its solubility in cell culture media or buffers before *in vitro* studies may be carried out. Approaches that have been implemented to deliver lycopene to cells in culture have included the use of organic solvents such as tetrahydrofuran, liposomes, emulsifiers, lipoproteins in fetal bovine serum, and water-dispersible beadlets. The addition of lycopene in such different modalities may induce considerable variations between laboratories in terms of efficacy of lycopene concentrations and incorporation into the cells.

## 3. Antitumoral Effect of Lycopene in Lung Tumorigenesis in Animal Models

Multiple animal models including rats, mice and ferrets, have been used to explore the efficacy of lycopene in the prevention of cancer in various tissues. Since the strongest clinical evidence for the benefits of lycopene in cancer are for prostate cancer chemoprevention, the majority of animal studies with lycopene have concerned prostate cancer. However, some studies to evaluate the efficacy of lycopene in lung tumorigenesis have been also conducted. The concentration of lycopene in rat lung (0.32 nmol/g) reported by Boileau *et al.* [62], following lycopene supplementation, was similar to that found in humans (0.6 nmol/g) [63]. The absorption of lycopene was shown to be dose-dependent in both male and female rats [63]. Lycopene concentrations have been reported to be highest in rat liver (120–42 μg/g wet wt.); physiologically significant levels have been also detected in rat prostate (97–47 ng/g), lung (227–134 ng/g), mammary gland (309–174 ng/g) and serum (285–160 ng/mL) [63]. A dose-dependency absorption of lycopene was also found in plasma and lungs of ferrets following lycopene supplementation [64]. In this study, the concentration of plasma lycopene (range from 226 to 373 nmol/L) in the ferret after lycopene supplementation was similar to the lycopene concentration (range, 290–350 nmol/L) reported in humans [65,66]. Furthermore, the lycopene concentrations in the lungs of ferrets that were given a low dose of lycopene (equivalent to 15 mg/day in humans) reached 342 nmol/kg, which is within the range of lung lycopene concentration in normal humans (100–500 nmol/kg) [4] suggesting a similarity between humans and ferrets with respect to lycopene absorption and accumulation. It has been also reported that lycopene concentration in ferrets supplemented with a high dose of lycopene (equivalent to 60 mg/day in humans) increased 3.4-fold in lung tissue and 1.6-fold in plasma, compared with those in ferrets supplemented with a low dose of lycopene (equivalent to 15 mg/day in humans). The fact that a greater increase in lycopene concentrations occurred in lung tissue than in plasma after lycopene supplementation should be considered in the design of future studies of lycopene supplementation.

Smoke exposure decreased the elevated lycopene concentrations in plasma and lung tissue of ferrets supplemented with lycopene [64], which is consistent with the data from National Health and Nutrition Examination Survey III that has found that smokers had lower serum level of lycopene compared with non-smokers [67]. Although all *trans*-lycopene is the predominant isomer in plasma and lung tissues of ferrets supplemented with lycopene (followed by 13-*cis*-lycopene and 9-*cis*-lycopene), smoke exposure appears to increase the *cis* isomers and decrease the *trans*-isomers of lycopene in the lungs of ferrets [64].

Both low- and high-dose lycopene supplementations substantially inhibited smoke-induced squamous metaplasia and proliferating cell nuclear antigen (PCNA) expression in the lungs of ferrets [64]. No squamous metaplasia or PCNA overexpression were found in the lungs of control ferrets or those supplemented with lycopene alone. Ferrets supplemented with lycopene and exposed to smoke had significantly higher plasma insulin growth factor binding protein 3 (IGFBP-3) levels and a lower insulin growth factor-1 (IGF-1)/IGFBP-3 ratio than ferrets exposed to smoke alone. The elevated phosphorylation of Bad and down-regulated apoptosis induced by cigarette smoke in the lungs of ferrets was prevented by both low- and high-dose lycopene supplementations. The work from Liu *et al.* [64] has important implications for future studies regarding chemopreventive effects of carotenoids against cancer, particularly for lung cancers. Using the ferret model, they have been demonstrated that the potential mechanisms for the harmful effects of high-dose β-carotene supplementation in smokers observed in human clinical trials may be due to the production of undesirable oxidative metabolites of β-carotene in the lung tissue [65,66]. The formation of oxidative by products from β-carotene can induce cytochrome p450 enzymes and interfere with retinoic acid metabolism [66,67] as well as down-regulate retinoic acid receptor (RAR)β [68]. In the ferrets supplemented with β-carotene at a dose of 30 mg/day, the concentration of β-carotene in the lungs of ferrets was 26 μmol/kg lung tissue, which was associated with an enhanced development of lung squamous metaplasia induced by cigarette smoke exposure [66]. In the lycopene study, the concentration of lycopene in the lungs was only 1.2 μmol/kg lung tissue in ferrets supplemented with lycopene at a dose of 60 mg/day, which caused no harmful effect but rather prevented the development of lung squamous metaplasia and cell proliferation induced by smoke exposure. The different outcome between the lycopene and β-carotene studies in ferrets may be attributable to the differences in the levels of carotenoids that accumulated in lung tissue. Therefore, from these observations, the inclusion of target tissue lycopene concentration, before and after treatment, is likely to provide a better understanding of the role of lycopene in the aetiology of human cancer.

Dietary lycopene dissolved in drinking water at a 50 ppm dose also significantly decreased diethylnitrosamine (DEH)-, methylnitrosourea (MNU)- and dimethylhydrazine (DMD)-induced lung adenomas along with carcinomas in male mice [69]. However, the protective effects of lycopene against lung cancer were not observed in female mice. Similarly, dietary lycopene did not alter colon or kidney tumors in the same study [69], suggesting a specificity of organ. However, in another study, treatment with dietary lycopene as lycopene-enriched tomato oleoresin (167, 1667 or 8333 ppm) from 1 week before carcinogen treatment until termination had no effect on tobacco-smoke carcinogens benzo[a]pyrene and 4-[methyl]nitrosamino]-1-[3-pyridyl]-1-butanone induced-lung tumor multiplicity in A/J mice [70]. Lack of protection against benzopyrene-induced lung tumors was probably due to the lack of involvement of DNA oxidation in this model [71]. Recently, the effects of tomato extract on acetaminophen (APAP), amiodarone (ADN) and cyclosporine A (CsA)-induced liver, lung and kidney toxicity have been studied [72]. Rats received a single dose of APAP before treatment with tomato extract (5 mg/kg, oral) for seven consecutive days, ADN plus tomato extract (5 mg/kg, oral) for 10 consecutive days, or CsA plus tomato extract (5 mg/kg, oral) for 14 consecutive days. Simultaneous treatment of tomato extract ameliorated tissue damage, biochemical indices, and oxidative stress parameters against APAP-induced acute toxicity, but had less beneficial effects on ADN-induced lung toxicity and little effect against CsA-induced nephrotoxicity.

In a recent issue of The Journal of Nutrition, Huang *et al.* [73] reported that lycopene inhibits experimental metastasis of human hepatoma SK-Hep-1 cells in athymic nude mice. In that study, lycopene or β-carotene-treated mice were injected with human hepatoma SK-Hep-1 cells via the tail vein. At the end of the experiment, lycopene-treated mice had a lower number of tumors and decreased tumor cross-sectional areas in the lung than the control mice. Lycopene treatment also decreased the rate of proliferating cell nuclear antigen, level of vascular endothelial growth factor, and protein expression of proliferating cell nuclear antigen, level of vascular endothelial growth factor, and metalloproteinase. Similar results were found in mice treated with β-carotene. Based on the data presented, β-carotene appears to be more effective than lycopene in attenuating the lung metastasis and related indices examined.

## 4. Mechanisms of Cancer Prevention by Lycopene

**Figure 2.**
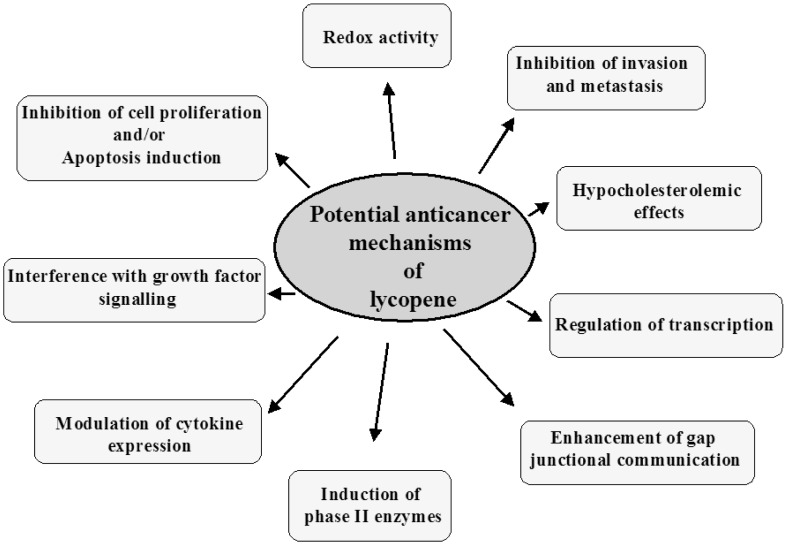
Potential mechanisms of lung cancer prevention by lycopene.

The potential mechanisms of lung cancer prevention by lycopene are described below and summarized in Figure 2.

### 4.1. Redox Activity

Oxidative stress is recognized as one of the major contributors to the increased risk of cancer. Currently, much work has been directed at characterizing the antioxidant capacity of lycopene. The system of conjugated double bonds allows lycopene molecule to efficiently quench the energy from very deleterious forms of oxygen (singlet oxygen) and to scavenge a large spectrum of free radicals. Lycopene has been reported to deactivate *in vitro* an array of free radicals, such as hydrogen peroxide, nitrogen dioxide, thyl, and sulphonyl [74-76]. There are a number of investigations demonstrating *in vitro* that lycopene is a more potent ROS scavenger than many other dietary carotenoids and other antioxidants, including vitamin E, and the rate constant of quenching singlet oxygen for lycopene is almost double that of *β*-carotene [30,40,43,77,78]. Woodall *et al.* [79] also reported that among all the tested carotenoids lycopene reacted most efficiently with peroxyl radicals generated by the thermal decomposition of azocompounds. The ability of lycopene to act as an antioxidant seems to depend on several factors, such as carotenoid concentration, type of oxidants involved in the oxidation reaction and interactions with other antioxidants. In a recent study, Lowe and colleagues [80] found that oxidative DNA damage caused by xanthine/xanthine oxidase was protected by low concentrations of lycopene (1–3 μM), but increased by higher concentrations (4–10 μM). Moreover, Yeh and Hu [81], in human foreskin fibroblasts, reported that lycopene significantly inhibited lipid peroxidation induced by ferric nitrilotriacetate, while it was ineffective in inhibiting lipid peroxidation induced by the water-soluble radical generator (2,2′-azobis(2-amidinopropane)dihydrochloride). Mixtures of carotenoids were more effective than the single compounds [82]. This synergistic effect was most pronounced when lycopene or lutein was present. The superior protection of mixtures may be related to the specific positioning of different carotenoids in cell membranes. Moreover, evidence is accumulating to suggest that lycopene may act as a modulator of intracellular ROS and, therefore, may control ROS-mediated cell growth [83]. The carotenoid has been reported to modulate redox-sensitive molecular targets involved in cell growth signaling such as antioxidant response elements, redox-sensitive mitogen-activated protein kinases (MAPKs), and transcription factors, including nuclear factor-kappaB (NF-kB) and activator protein-1 (AP-1).

Despite these promising antioxidant effects of lycopene *in vitro*, at the moment, there is limited support for an *in vivo* antioxidant role of lycopene, as recently reviewed by Erdman and colleagues [84]. This is due to several factors, including the limited number of studies, the extreme variability of lycopene administration in terms of dose and modality, the difficulty to find out adequate methods for testing antioxidant activity, the possibility of interactions among lycopene and other bioactive food components, which may affect lycopene uptake, metabolism and stability.

### 4.2. Inhibition of Cancer Cell Proliferation and Apoptosis Induction

Lycopene has been found to inhibit proliferation of several types of cancer cells. The inhibitory effects of lycopene have been accompanied by inhibition of cell cycle progression from the G0/G1 to the S phase [85] and by changes in proteins controlling cell cycle. In particular, lycopene has been reported to decrease cyclin D1 and to increase p53, p21^Waf1/Cip1^ protein levels in cancer cells.

The cancer-preventive effect of lycopene mediated by its ability to induce apoptosis has been also reviewed [86]. The carotenoid has been reported to modulate Bcl-2, Bad, Bid and Bax expression and/or phosphorylation in different experimental models.

The Ku proteins are involved in multiple cellular pathways, including DNA repair, telomere maintenance and Bax-mediated apoptosis. Recently, lycopene has been reported to reduce the levels of H_2_O_2_, to arrest cell cycle progression and to inhibit Ku-DNA binding activity, and cellular and nuclear levels of Ku70 in Pancreatic Acinar AR42J cells [87]. These data suggest that the carotenoid may be beneficial for the treatment of oxidative stress-induced cell death by preventing loss of DNA repair protein Ku70.

### 4.3. Interference with Growth Factors Stimulation of Cancer Cell Proliferation

Increasing evidence suggests that lycopene may modulate IGF-1 pathway, reducing IGF-1-stimulated cell growth in cancer cell lines [28] decreasing IGF-1, IGF-1R, or increasing IGFBP-1 or IGFBP-3 in animal studies [64,88,89] and in human subjects [90-92]. Sharoni *et al.* [93,94] provided a potential mechanism whereby lycopene interfered with IGF-1-stimulated cell growth. They showed that IGF-1-stimulated cell growth, as well as DNA binding activity of the AP-1 transcription factor, were reduced by physiological concentrations of lycopene in endometrial, mammary, and lung cancer cell lines. In such models, lycopene was able to inhibit IGF-1-stimulated insulin receptor substrate 1 phosphorylation and cyclin D1 expression, to block IGF-1-stimulated cell cycle progression [95] and to increase membrane-associated IGFBPs [95,96]. It has also been suggested that cigarette smoke exposure may promote cell proliferation and neoplasia by affecting normal IGF-1 signaling [96]. Recent studies seem to demonstrate interactions of lycopene with smoke by a direct mechanism involving IGF-1/AKT pathways. In the lung of ferrets, it has been shown that cigarette smoke-induced lesions (e.g., squamous metaplasia, PCNA over-expression, and diminished apoptosis) were associated with reduced plasma IGFBP-3 concentrations and increased IGF-1/IGFBP-3 ratios [64]. Such changes significantly affected the status of cell proliferation and apoptosis in the lung of ferrets. Smoke exposure significantly decreased cleaved caspase-3 protein and increased PCNA. Furthermore, smoke exposure suppressed Bad-mediated apoptosis by inducing the phosphorylation of Bad at both Ser^136^ and Ser^112^. These smoke-induced changes were prevented by lycopene supplementation in a dose-dependent manner. The carotenoid was able to increase IGFBP-3 levels, and to decrease IGF-1/IGFBP-3 ratio. Moreover, it decreased Bad phosphorylation at both Ser^136^ and Ser^112^ and increased cleaved caspase-3, preventing cigarette smoke-induced squamous metaplasia and the increase in PCNA [64]. A recent *in vitro* study also suggests that the modulation of AKT pathway may have a key role in the pro-apoptotic effects of lycopene under smoke conditions [97]. In fact, while RAT-1 fibroblasts exposed to cigarette smoke condensate (tar) alone exhibited high levels of phosphorylated AKT, cells exposed to a combination of tar and lycopene strongly decreased them. Moreover, the exposition of RAT-1 fibroblasts to tar alone suppressed Bad-mediated apoptosis by inducing the phosphorylation of Bad at Ser^136^ [97]. Conversely, lycopene was able to completely prevent the phosphorylation of Bad induced by tar, confirming *in vitro* the results obtained *in vivo* by Liu *et al.* [64].

### 4.4. Cancer Prevention by Inducing Phase II Enzymes

Induction of phase II enzymes, which conjugate reactive electrophiles (chemicals that are attracted to electrons or tend to accept electrons from other chemicals) and act as indirect antioxidants, appears to be an effective means for achieving protection against a variety of carcinogens in animals and humans. Bhuvaneswari *et al.* [98] associated the chemopreventive (cancer-preventive) effect of lycopene on the incidence of DMBA-induced hamster buccal (cheek, mouth) pouch tumors with a simultaneous rise in the level of reduced glutathione, enzymes of the glutathione redox cycle, and glutathione S-transferase (GST) in the buccal pouch mucosa. (DMBA is a 9,10-dimethylbenz-α-anthracene, a potent tumor-initiating compound.) These results suggest that the lycopene-induced increase in the levels of GSH and the phase II enzyme GST inactivates carcinogens by forming conjugates (chemicals formed by two or more compounds), products that are less toxic and readily excreted. Astorg and colleagues [99] proposed that lycopene-induced modulation of the liver metabolizing enzyme, cytochrome P4502E1, was the underlying mechanism of protection against carcinogen- induced preneoplastic lesions in the rat liver. Moreover, the administration of lycopene to rats was shown to induce liver CYP types 1A1/2, 2B1/2 and 3A in a dose-dependent manner [100]. Recently, both lycopene and β-carotene have been shown to activate the cytochrome P450 1A1 gene but only β-carotene was able to induce the retinol dehydrogenase gene in mice [101].

### 4.5. Regulation of Transcription

Transcription is the process whereby genetic information is carried from the DNA molecule via the RNA molecule acting as a messenger. This biochemical route leads to the formation of new proteins by the process called translation. As discussed above, lycopene modulates the basic mechanisms of cell proliferation, growth factor signaling, and gap junctional intercellular communication [102]. Additionally, lycopene produces changes in the expression of many proteins participating in these processes, e.g., connexins, cyclins, and phase II enzymes. Therefore, the question that arises is “By what mechanisms does lycopene affect so many diverse cellular pathways?” The changes in the expression of multiple proteins suggest that the initial effect of lycopene involves modulation of transcription; this process is reviewed by Sharoni *et al.* [103] in a recent publication. This may be due to either direct interactions of the carotenoid molecules or their derivatives with transcription factors (e.g., with ligand-activated nuclear receptors [104] or indirect modifications of transcriptional activity (e.g., via changes in status of cellular redox, which affects redox-sensitive transcription systems [83].

### 4.6. Hypocholesterolemic Effects

Cancer cells have abnormal cholesterol biosynthetic pathways that are resistant to down-regulation by cholesterol, and farnesylation is a key process in oncogene activation [105,106]. Both β-carotene and lycopene share similar initial synthetic pathways with cholesterol, which is synthesized in animal but not in plant cells. In a recent paper, it has been tested the hypothesis that lycopene may exert its antitumor effects through changes in mevalonate pathway and in Ras activation [56]. In different tumor cell lines, including lung BEN cancer cells, lycopene treatment dose-dependently reduced intracellular total cholesterol by decreasing 3-hydroxy-3-methylglutaryl-coenzyme A (HMG-CoA) reductase expression. Such an effect was accompanied by Ras inactivation, as evidenced by the translocation of the protein from cell membranes to cytosol and by an arrest of cell cycle progression and apoptosis induction [56].

### 4.7. Modulation of Cytokine Expression

Pro-inflammatory cytokines, such as interleukins (ILs) and tumor necrosis factor-alpha (TNF-α), have been implicated in tumor promotion in various experimental models of tumorigenesis. The lycopene potential ability to influence cytokine levels may be at least in part explained by carotenoid localization in or within cell membrane, modulating surface molecules for primary immune response, ROS production, the activity MAPKs and transcription factors, such as NF-κB. The modulation of pro-inflammatory cytokine levels by lycopene and/or tomato products has been recently reviewed [33].

### 4.8. Enhancement of Gap Junctional Communication

Both carotenoids and retinoids stimulate gap junction communication (GJC) through stabilization of connexin43 mRNA. Because GJC is lost in cancer cells, its restoration is considered to be a cancer-preventive property of carotenoids and retinoids. If lycopene is cleaved by analogy to the conversion of β-carotene to retinoic acid, then acycloretinoic acid is formed. Both lycopene and this cleavage product, which could result from oxidation, were tested *in vitro* for their effect on GJC, on stabilization of connexin43 mRNA, and on the transactivation of the RAR-β2-promoter by Stahl *et al.* [104]. In human fetal skin fibroblasts, GJC was stimulated by lycopene and acyclo-retinoic acid. Lycopene was effective at a concentration of 0.1 μM, whereas higher amounts of acyclo-retinoic acid (1 μM) were needed for comparable stimulation. Stabilizing effects of acycloretinoic acid on the mRNA of connexin43 via elements located in the 3μ-un-translated region were weak. In comparison with retinoic acid (0.1 μM), considerably higher concentrations of the acyclo analog (50 μM) were required for similar effects; lycopene (0.1 μM) was not active in this system. Likewise, un-physiologically high levels of acycloretinoic acid (50 μM) were necessary to transactivate the RAR-β2 promoter. The data demonstrate that acycloretinoic acid is much less active than retinoic acid with respect to GJC and retinoid-related signaling. These data are consistent with the conclusion that lycopene affects GJC independent of the formation of acyclo-retinoic acid. In fact, it is not established that acyclo-retinoic acid is an important physiologically active oxidation product of lycopene in humans. Therefore, although it is still possible that lycopene may act through the RAR receptor mechanism, much more research is needed on this question.

### 4.9. Inhibition of Invasion and Metastasis

One critical characteristic that metastatic cancer cells have acquired is the ability to dissolve basement membranes and the extracellular matrix (ECM). This degradative process is mediated largely by matrix metalloproteinases (MMPs), which are a large family of, at least, 20 zinc-dependent neutral endo-peptidases that together can degrade all known components of ECM. MMP-9 is abundantly expressed in various malignant tumors and is postulated to play a critical role in tumor invasion and angiogenesis. In studies of the highly invasive human hepatoma cell line SK-Hep-1, lycopene was shown to have anti-metastatic and anti-invasion activity. Hwang and Lee [60] showed that lycopene at 5 and 10 μM (higher than physiologically relevant concentrations) could decrease the gelatinolytic activities of the matrix metalloproteinases MMP-2 and MMP-9 and inhibit the adhesion, invasion and migration of SK-Hep1 cells. At similar lycopene concentrations, Huang *et al.* [59] confirmed that MMP-9 expression was suppressed in SK-Hep-1 cells and found that the metastasis suppressor gene nm23-H1 was induced. These studies indicate that lycopene can inhibit metastasis and invasion by hepatocarcinoma cells, although at high carotenoid concentrations that might not be physiologically attainable.

Platelet-derived growth factor (PDGF) functions as a mitogen for dermal fibroblast chemotaxis [107] and can stimulate tumor angiogenesis [108]. Since PDGF-BB facilitates the growth, invasion and metastasis of melanoma, inhibition of these PDGF-BB effects can be a mechanism for arresting melanoma progression. *In vitro* studies with the human foreskin fibroblast cell line Hs68 and the human metastatic melanoma cell line A2058 both individually and in a co-culture system indicate that lycopene can inhibit PDGF-BB induced human Hs68 skin fibroblast migration, attenuate PDGF-BB induced phosphorylation, and reduce PDGF-BB induced signaling [109]. In addition, lycopene was shown to bind to PDGF-BB in human plasma. Therefore, these *in vitro* studies suggest that lycopene might control the progression of melanoma.

## 5. Role of Lycopene Metabolites in Tumorigenesis

Several lycopene metabolites have been identified *in vitro* [110-112] and *in vivo* [112-115] systems, raising the question as to whether the preventive effects of lycopene on cancer risk is, at least in part, due to its metabolites [116].

Previously, Ben-Dor *et al.* showed that an ethanolic extract of lycopene containing unidentified hydrophilic derivatives induced phase II enzymes and activated ARE-driven reporter gene activity at a potency similar to lycopene [116]. Moreover, lycopene metabolites were found to mediate the activation of the antioxidant response element transcription system [117]. The carotene 9′,10′-oxygenase, a specific cleavage enzyme at the 9′,10′ double bonds of carotenoids, has been cloned from humans, rats, mice and ferrets [115] It has been recently demonstrated that the enzymatic cleavage of lycopene at its 9′,10′-double bond by carotene 9′,10′-oxygenase produces apo-10′-lycopenal, which can be either reduced into apo-10′-lycopenol or oxidized into apo-10′-lycopenoic acid [115]. Very recently, it has been shown that apo-10′-lycopenoic acid treatment for 16 weeks suppresses chemical carcinogen (4-(*N*-methyl-*N*-nitrosamino)-1-(3-pyridal)-1-butanone, NNK)-induced lung tumorigenesis in the A/J mouse model [118]. Although *in vitro* experiments showed that apo-10′- lycopenoic acid inhibits lung cancer cell growth and activates retinoic acid receptor signaling [118], the corresponding effects were not observed in the lungs of the mice after 16 weeks of treatment. These results suggest that, in addition to its growth inhibitory activity, apo-10′-lycopenoic acid may provide protection against the initiation stage of carcinogenesis, e.g., detoxifying NNK or counteracting oxidative insults via its induction of phase II detoxification/antioxidant enzymes.

Moreover, it has been reported that treatment with apo-10′-lycopenoic acid, in a time- and dose-dependent manner, resulted in the nuclear accumulation of transcription factor Nrf2 (nuclear factor E2-related factor 2) protein in BEAS-2B human bronchial epithelial cells [119]. The activation of Nrf2 by apo-10′-lycopenoic acid was associated with the induction of phase II detoxifying/antioxidant enzymes including heme oxygenase-1, NAD(P)H:quinone oxidoreductase 1, glutathione S-transferases, and glutamate-cysteine ligases in BEAS-2B cells. Furthermore, apo-10′-lycopenoic acid treatment increased total intracellular glutathione levels and suppressed both endogenous reactive oxygen species generation and H_2_O_2_-induced oxidative damage in BEAS-2B cells. In addition, both apo-10′-lycopenol and apo-10′-lycopenal induced heme oxygenase-1 gene expression in BEAS-2B cells. These data strongly suggest that the anti-carcinogenic and antioxidant functions of lycopene may be mediated by apo-10′-lycopenoids via activating Nrf2 and inducing phase II detoxifying/antioxidant enzymes.

## 6. Lycopene and Lung Cancer: Observation Epidemiology

Several case-control and cohort studies have examined the interaction existing between lycopene and/or tomato products and lung cancer risk. At the moment, the results are quite controversial. Many case-control studies have suggested reduced risk of lung cancer with higher lycopene intakes [120-124]. However, some cohort study findings have typically been null [125-130]. In particular, Mannisto *et al.* [125], using data from seven cohort studies in the United States and Europe, reported that none of the carotenoids evaluated in the study, including lycopene, was associated with lung cancer risk. In the VITAL Study, no significant association of supplemental lycopene with lung cancer risk was observed [131]. On the other hand, in the Health Professionals Follow-up Study and the Nurses' Health Study, lycopene and total carotenoids (from diet) were associated with reduced lung cancer risk. Even in the ATBC cohort [129], lower risks of lung cancer were reported for the highest *versus* the lowest quintiles of self-reported dietary intakes of total carotenoids and lycopene, as well as serum β-carotene and serum retinol. The differences among the studies may be due to the heterogeneity of tomato lycopene and/or tomato products consumption, to decreased attention in quantitative dietary assessment, to measurement error or to differences in bio-availability assessment.

A recent review has examined the evidence for the associations between carotenoids, including lycopene, and the risk of lung cancer [132]. Such a study, carried out as a part of a project funded by the World Cancer Research Fund (WCRF) and the American Institute for Cancer Research, has evaluated the results of cohort studies analyzing the association between dietary intake of carotenoids and lung cancer [133-145] and cohort studies investigating the association between serum or plasma carotenoid concentrations and lung cancer [128,129,134, 146-157]. The results of this report provide evidence that, for individual carotenoids, the associations remain weak and statistically non-significant, with the only exception for lycopene. In both dietary intake and serum studies, the association between lycopene and lung cancer was statistically significant in the protective direction. However, also for lycopene, dose-response trends were observed only in serum studies. In contrast, for total carotenoids, the results provided stronger and more consistent evidence for an inverse association and statistically significant dose-response trends were present in both the dietary intake and serum studies.

Even these positive results, caution in conclusions is needed. In fact, these inverse associations may be the result of carotenoid measurements' function as a marker of a healthier lifestyle (higher fruit and vegetable consumption) or of residual confounding by smoking. Since the strong association between smoking and lung cancer and the changes in carotenoid intake and consumption between smokers and non-smokers, smoking status seems to be an effective modifier for lycopene effects in lung cancer risk [125,132]. The WCRF report evidence that β-carotene supplements cause lung cancer in current smokers, and also that foods containing carotenoids protect against lung cancer [158]. However, the report from Gallicchio *et al.* [132], selectively focused on prospective studies adjusted for cigarette smoking, suggests that residual confounding by smoking remains a possible explanation for the associations between carotenoids and lung cancer seen in the observational studies. In fact, in analyses restricted to never smokers, the association between β-carotene intake and lung cancer was null. On the other hand, the intake of carotenoids other than β-carotene tended to be inversely associated with lung cancer among both never and current smokers.

## 7. Conclusions

*In vitro* studies, despite their relatively artificial nature, demonstrated that lycopene may inhibit the growth of lung cancer cells and provided valuable insights into the mechanisms by which carotenoids, such as lycopene exert their cellular and intracellular effects. Various mechanisms have been proposed to explain the inhibitory effects of lycopene, including cell cycle arrest and/or apoptosis induction via a modulation of redox status, a regulation of growth factor signaling, changes in cell growth-related enzymes, an enhancement of gap junction communication, and a prevention of smoke-induced inflammation. In addition, lycopene also inhibited cell invasion, angiogenesis, and metastasis. Several lycopene metabolites have been identified, raising the question as to whether the preventive effects of lycopene on cancer risk is, at least in part, due to its metabolites, although none has been proven definitively. Cell culture studies have the disadvantage that the concentrations of lycopene used are often supra physiological and that *in vitro* the O_2_ partial pressures are higher than under *in vivo* conditions. Another problem with *in vitro* approaches lies in the extreme lipophilicity of lycopene. Approaches that have been implemented to deliver lycopene to cells in culture have included the use of organic solvents such as tetrahydrofuran, liposomes, emulsifiers, lipoproteins in fetal bovine serum, and water-dispersible beadlets. The addition of lycopene in such different modalities may induce considerable variations between laboratories in terms of efficacy of lycopene concentrations and incorporation into the cells.

There are relatively few reports on lung cancer chemopreventive effects of lycopene or tomato carotenoids in animal models. A good number, but not all, of these studies indicates a protective effect on lung tumorigenesis. Unfortunately, because of differences in routes of administration (gavage, intraperitoneal injection, intra-rectal instillation, drinking water, and diet supplementation), species and strain differences, form of lycopene (pure crystalline, beadlet, mixed carotenoid suspension), varying diets (grain-based, casein based) and dose ranges (0.5–500 ppm), no real comparison is possible among the different studies. It is clear that the majority of ingested lycopene is excreted in the feces and that 1000-fold more lycopene is absorbed and stored in the liver than accumulated in other target organs. Nonetheless, physiologically significant (nanogram) levels of lycopene are assimilated by key organs, including lung tissue and there is a rough dose-response relationship between lycopene intake and blood levels. Pure lycopene is absorbed less efficiently than the lycopene-rich tomato carotenoid oleoresin and blood levels of lycopene in rats fed a grain based diet were consistently lower than those in rats fed lycopene in a casein-based diet. The latter suggests that the matrix in which lycopene is incorporated is an important determinant of lycopene uptake.

Many case-control and cohort studies have examined lycopene-rich diets and lung cancer, suggesting an association between lycopene and lung cancer in the protective direction. However, the inverse association between lycopene intake and risk of lung cancer may be the result of carotenoid measurements' function as a marker of a healthier lifestyle (higher fruit and vegetable consumption) or of residual confounding by smoking. Moreover, a number of issues remain to be resolved before any definitive conclusions can be drawn concerning the preventive effects of lycopene in lung cancer. These include the following: the optimal dose and form of lycopene, interactions among lycopene and other carotenoids and fat soluble vitamins, the role of dietary fat in regulating lycopene uptake and disposition, organ and tissue specificity, and the problem of extrapolation from animal models to human populations. There is a great need for well-designed human intervention studies that take into consideration study designs including subject selection, specific markers of analysis, the levels of carotenoids being tested, metabolism and isomerization of lycopene and their biological significance. It is only through such studies that our understanding of the anticancer role played by tomato lycopene will be enhanced and help us to develop complementary strategies for the prevention, treatment and management of lung cancer.
